# Imputation of missing genotypes within LD-blocks relying on the basic coalescent and beyond: consideration of population growth and structure

**DOI:** 10.1186/s12864-017-4208-2

**Published:** 2017-10-17

**Authors:** Maria Kabisch, Ute Hamann, Justo Lorenzo Bermejo

**Affiliations:** 1 Molecular Genetics of Breast Cancer, German Cancer Research Center (DKFZ), 69120 Heidelberg, Germany; 2 Institute of Medical Biometry and Informatics, University of Heidelberg, 69120 Heidelberg, Germany

**Keywords:** Genotype imputation, Coalescent theory, Linkage disequilibrium, Imputation accuracy, Population growth, Population structure

## Abstract

**Background:**

Genotypes not directly measured in genetic studies are often imputed to improve statistical power and to increase mapping resolution. The accuracy of standard imputation techniques strongly depends on the similarity of linkage disequilibrium (LD) patterns in the study and reference populations. Here we develop a novel approach for genotype imputation in low-recombination regions that relies on the coalescent and permits to explicitly account for population demographic factors.

To test the new method, study and reference haplotypes were simulated and gene trees were inferred under the basic coalescent and also considering population growth and structure. The reference haplotypes that first coalesced with study haplotypes were used as templates for genotype imputation. Computer simulations were complemented with the analysis of real data. Genotype concordance rates were used to compare the accuracies of coalescent-based and standard (IMPUTE2) imputation.

**Results:**

Simulations revealed that, in LD-blocks, imputation accuracy relying on the basic coalescent was higher and less variable than with IMPUTE2. Explicit consideration of population growth and structure, even if present, did not practically improve accuracy. The advantage of coalescent-based over standard imputation increased with the minor allele frequency and it decreased with population stratification. Results based on real data indicated that, even in low-recombination regions, further research is needed to incorporate recombination in coalescence inference, in particular for studies with genetically diverse and admixed individuals.

**Conclusions:**

To exploit the full potential of coalescent-based methods for the imputation of missing genotypes in genetic studies, further methodological research is needed to reduce computer time, to take into account recombination, and to implement these methods in user-friendly computer programs. Here we provide reproducible code which takes advantage of publicly available software to facilitate further developments in the field.

**Electronic supplementary material:**

The online version of this article (10.1186/s12864-017-4208-2) contains supplementary material, which is available to authorized users.

## Background

The imputation of missing genotypes is a commonly used technique to increase the statistical power of genetic association studies and to fine-map causal variants [1]. Genotype imputation strongly relies on linkage disequilibrium (LD) and, since recombination breaks allelic association, imputed genotypes mainly depend on genotypes measured within the same LD-block. Several methods have been developed to impute genotypes, most of them rely on current, observed LD patterns. For example, IMPUTE2 is a well-established and accurate program for genotype imputation [2, 3]. It estimates the posterior probability of missing genotypes in a study individual by conditioning on the estimated haplotype of the study individual, on the estimated haplotypes of individuals in an external reference panel for genotype imputation, and on an external recombination map. As a result, the similarity of LD patterns in the study and the reference populations is crucial for a precise genotype imputation.

The accuracy of imputed genotypes is influenced by several factors, e.g. the density of measured variants in the study population and their physical location in the genetic region of interest. Lack of reference haplotypes for rare and low-frequency variants may lead to low imputation accuracy and false-positive associations. Huang et al. demonstrated that imputation accuracy is decreased for African populations in comparison to European, Asian and American populations due to the higher genetic diversity and the lower LD levels [4, 5]. Imputation accuracy is also determined by demographic factors. Marchini et al. stated that IMPUTE2 accuracy is decreased when population structure exists within the study and also as a result of differences in LD patterns between the study and reference populations [3]. For these reasons, genotype imputation may benefit from methods which incorporate a population genetics model to circumvent the dependence on LD, and to explicitly consider demographic factors such as population growth and structure.

Coalescence theory is essential when analyzing genetic variants sampled at the present time with a focus on evolutionary forces which shaped current genetic variability [6, 7]. Modeling the past can be done by a stochastic process denominated *the coalescent*, which can be interpreted as the genealogical view of mutation backward in time. In a gene tree, sampled gene copies are related to each other through coalescence events, i.e. two distinct genetic lineages coalesce into one shared ancestral lineage. The essential idea of coalescent-based genotype imputation is to exploit the properties of the population’s gene genealogy in order to identify haplotypes from the reference panel that can adequately function as imputation templates for study haplotypes.

The aim of the present investigation was to develop and test a novel imputation approach which relies on the coalescent and permits to explicitly account for population growth and structure. Instead of exclusively relying on current LD patterns, we capitalize on the coalescent process and estimate the underlying gene genealogies. The advantages and limitations of this alternative approach in comparison to standard genotype imputation were examined using simulated and real data.

## Methods

**Fig. 1:**
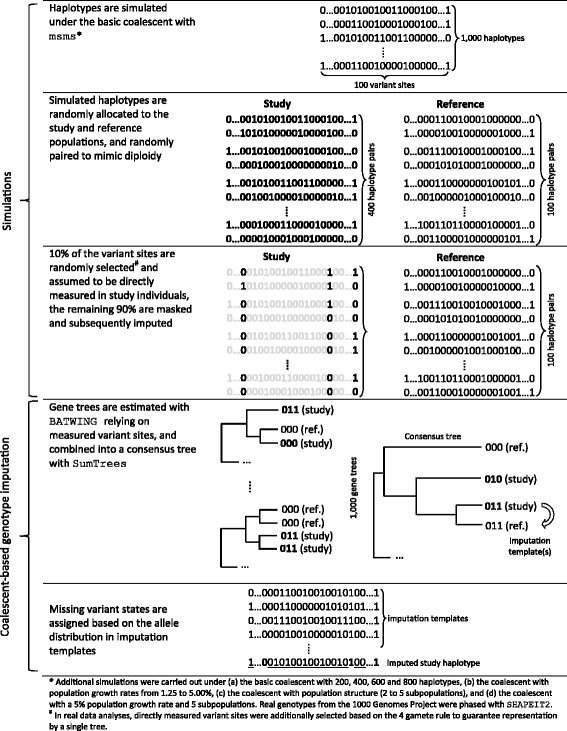
Overview of conducted simulations and proposed algorithm for coalescent-based genotype imputation

Computer simulations and real data were used to assess the potential and limitations of coalescent-based genotype imputation. Figure 1 provides an overview of the conducted simulations and the investigated coalescent-based imputation approach. In brief, haplotypes were simulated under the basic coalescent and two extensions thereof: population growth and population structure. After random allocation to the study and the reference population, haplotypes were arranged by chance in pairs to mimic diploidy. Directly measured variants in the study were randomly selected, and consensus gene trees were built without and with consideration of growth and structure. The reference haplotype(s) which first coalesced with a study haplotype were used as imputation template(s). In addition to simulation, coalescent-based genotype imputation was evaluated based on real data from the 1000 Genomes Project (1000 GP). Accuracy was measured by the concordance between true masked and imputed genotypes in both simulated and real data. The software IMPUTE2 was regarded as gold standard method for genotype imputation. The following sections describe in detail the implemented computations and the investigated real datasets. Table 1 provides background information about the programs and methods used in the present study. Computer code to reproduce all calculations is available through www.biometrie.uni-heidelberg.de/StatisticalGenetics/Software_and_Data.

**Table 1:** Brief background information about the programs and methods used in the present study

Program/Method	Background information	Application to the present study	Reference
BATWING	The program reads multi-locus haplotype data and uses a Markov chain Monte Carlo method based on coalescent theory to generate approximate random samples of the underlying gene genealogy. BATWING allows specification of the population growth and structure models with their corresponding prior distributions.	Estimation of gene genealogies underlying haplotypes under the basic coalescent and also considering population growth and structure	[12]
Genetree	The program constructs gene trees describing the history of a sample of DNA sequences and calculates maximum likelihood estimates of the time to the most recent common ancestor and mutation, migration and growth rates, also in substructured populations.	Exclusion of incompatible sites by pairwise four-gamete tests	[11]
IMPUTE2	Computer program for phasing observed genotypes and imputing missing genotypes. Basically, phasing and imputation are alternatively iterated in a Markov chain Monte Carlo framework which accounts for phase uncertainty.	Used as gold standard for genotype imputation assuming no recombination and also considering regional recombination rates	[3]
msms	Extension of Hudson’s coalescent simulator ms, which also permits to study selection. Since selection was not considered in the present study, our haplotypes were simulated using standard coalescent methods: genealogies were generated by tracing randomly sampled alleles backwards in time.	Haplotype simulation under the basic coalescent and also considering population growth and structure	[8]
SHAPEIT2	Fast and accurate method for phasing from genotype or sequencing data.	Phasing of real genotype data	[10]
SumTrees	The program constructs a summary tree based on tree samples provided by the user. Supported methods for summary tree construction include the Maximum Clade Credibility Topology, and the majority-rule clade consensus.	Combination of gene genealogies estimated by BATWING into a majority-rule consensus tree	[14]

### Haplotype simulations under the basic coalescent and two extensions

Under the baseline simulation scenario, 1000 haplotypes with 100 polymorphic sites each were generated under the basic coalescent without recombination using msms [8]. We fixed the effective population size to *N*_*e*_ = 10,000 and the mutation rate per site per generation to *μ* = 1.25 × 10^−8^. Assuming an average genetic variability of one single nucleotide polymorphism (SNP) every 200 base pairs for the human genome, simulated haplotypes covered deoxyribonucleic acid (DNA) segments of approximately 20 kilobases (kb) [9].

We hypothesized that study individuals were genotyped for a subset of variants in the region of interest, that reference individuals from an external data repository were genotyped for this subset and for additional variants, and that haplotypes were available for all study and reference individuals (Fig. 1). To emulate the study and the reference populations, 800 out of 1000 simulated haplotypes were randomly assigned to the study population and the remaining 200 haplotypes built the external reference panel. Within the study and the reference populations, haplotypes were randomly paired in order to mimic diploidy. Ten percent of the 100 variant sites were randomly selected and assumed to be directly measured in the study. Genotypes in the remaining sites were masked and subsequently imputed. Under the baseline simulation scenario, 10 replicates were generated using different seeds for haplotype simulation with msms, and 10 different seeds were used for the random selection of directly measured variants.

In addition to the baseline simulation scenario comprising 1000 haplotypes in total (800 study and 200 reference haplotypes), simulations were conducted for 800 (640 plus 160), 600 (480 plus 120), 400 (320 plus 80) and 200 (160 plus 40) haplotypes. The possible effect of population growth on imputation accuracy was examined considering different exponential growth rates (*α*). Besides a constant population size (*α* = 0%) in the baseline simulation scenario, four populations with 1000 haplotypes each were simulated assuming that exponential growth started 5000 years ago with rates 1.25, 2.50, 3.75 and 5.00%.

The possible effect of population structure on imputation accuracy was investigated by varying the number of subpopulations (*β*). The size of each subpopulation was fixed to 200 haplotypes. Four populations totaling 1000, 800, 600 and 400 haplotypes each which split 50,000 years ago into *β* = 5, 4, 3 and 2 equally large subpopulations, respectively, were simulated. The most complex simulated scenario simultaneously considered growth and structure. A population with 1000 haplotypes was assumed to split 50,000 years ago into *β* = 5 equal subpopulations, of which each started 5000 years ago to grow exponentially at rate *α* = 5.00%.

### Real genotypes from the 1000 genomes project

Real data were retrieved from the 1000 GP phase 1 [9]. Genotype imputation was carried out for three hypothetical studies. In the first hypothetical study, 85 CEU individuals built the study population and all remaining 1000 GP individuals constituted the external reference panel for genotype imputation. This scenario represents a study which includes persons from a relatively young and genetically homogenous population, and where genetically similar external reference subpopulations (GBR, FIN, IBS and TSI) are available. In the second hypothetical study, all African subpopulations (ASW, LWK and YRI comprising 246 individuals) built the study population, the remaining European, Asian and Latin American individuals belonged to the reference population. This scenario represents a study with individuals from an old and genetically diverse population, without available genetically similar reference panels. In the third hypothetical study, Latin American subpopulations CLM, MXL and PUR comprising 181 individuals mimicked the study of a population subject to recent growth and substructure related to genetic admixture.

Genotypes were imputed for a genetic region on chromosome 22, which was selected based on recombination rates from map GRCh37 provided by the 1000 GP. The region (28,252,000–28,272,000) showed a low average recombination rate (*ρ* = 0.05 centimorgan per megabase (cM/Mb). In contrast to simulation experiments where we directly generated haplotypes, the assessment of coalescent-based genotype imputation relying on real data required phasing, which was implemented using SHAPEIT2 [10]. In analogy with simulated data, 10 % of real genetic variants were assumed to be directly measured in the study, the genotypes of the remaining variants were masked and subsequently imputed. Measured genetic variants were randomly selected after exclusion of incompatible sites by pairwise four-gamete tests. Incompatible sites were excluded using a built-in feature of Genetree since this would imply recombination and the necessity to infer gene graphs instead of gene trees [11].

### Proposed algorithm for coalescent-based genotype imputation

The lower part of Fig. 1 illustrates the novel approach for genotype imputation, which consists of 1) the estimation of underlying gene genealogies, 2) the combination of gene genealogies into a majority-rule consensus tree, and 3) the estimation of allele probabilities relying on identified imputation templates.

Gene genealogies underlying simulated haplotypes were estimated with BATWING [12]. Note that genealogy estimation relied on variant sites which were assumed to be directly measured in the study (Fig. 1). Prior distributions were selected as suggested by Wilson and Balding [13]. In total, 1000 gene trees were generated per configuration assuming a uniform(0,100) prior distribution of the scaled mutation rate. The generation of 1000 gene trees was repeated 10 times under the baseline simulation scenario (basic coalescent). Gene genealogies were also estimated considering population growth and structure. Population growth was taken into account assuming that exponential growth started 5000 years ago and that the growth rate *α* had a gamma prior distribution (*Γ*(2160) for 1.25%, *Γ*(2,80) for 2.5%, *Γ*(2,53) for 3.75% and *Γ*(2,40) for 5.00%). Population structure was taken into account through the number of subpopulations, the splitting time and the proportion of the total population size taken up by each subpopulation (Dirichlet(*β*,2) distributed, where *β* was the number of subpopulations).

The 1000 gene trees inferred with BATWING were combined into a majority-rule consensus tree using SumTrees [14]. Thereafter, means of Bayesian posterior coalescence times were mapped onto tree branches. Imputation templates were identified based on the consensus tree as follows. If the reference panel included haplotypes identical to the study haplotype regarding directly measured variant sites, identical reference haplotypes were used as imputation templates. If no identical haplotype was present in the reference panel, the haplotype in the reference panel which first coalesced with the study haplotype was used as imputation template. If there were multiple haplotypes in the reference panel which coalesced with a given study haplotype not more than 0.001 coalescent units (10 generations) apart, all these reference haplotypes served as imputation templates. Allele probabilities of masked genetic variants were estimated relying on the distribution of alleles among selected imputation templates.

### Comparison of standard and coalescent-based genotype imputation

Genotypes were imputed relying not only on the coalescent but also using IMPUTE2 [3]. IMPUTE2 was selected as gold standard because it is one of the most accurate imputation methods and because it is widely applied [2]. IMPUTE2 estimates the posterior probability of each study individual’s unmeasured genotype conditioned on its estimated haplotype, the estimated haplotypes of reference individuals and an external recombination map. Let assume L diallelic SNPs with alleles coded as 0 and 1. Let H be a set of N known haplotypes at the L SNPs, and let G be a set of genotypes at the same L SNPs in K individuals with G_i_ = {G_i1_, …, G_iL_} denoting the genotypes of the ith individual. Individual genotypes are either measured so that G_ik_ ∈ {0, 1, 2}, or they are missing. To predict genotypes which have not been measured in study individuals, IMPUTE2 uses a hidden Markov model of each individual’s genotype vector G_i_, conditional on H, and a set of parameters:

P(G_i_| H, µ, ρ) = ∑_*Z*_P(G_i_| Z, µ), P(Z| H, ρ),

where Z = {Z_1_, …, Z_L_} with Z_j_ = {Z_j1_, Z_j2_} and Z_jk_ = {1, …, N}. Here, Z_j_ is the pair of haplotypes from the reference at SNP j that are being copied to form the genotype vector. P(Z| H, ρ) models the change of copied haplotypes along the sequence and is defined by a Markov chain in which switching between states depends on a recombination map with recombination rate ρ measured in cM/Mb and scaled by 4N_e_. P(G_i_| Z, µ) allows each measured genotype vector to differ through mutation from the genotypes determined by the pair of copied haplotypes and is controlled through the mutation parameter μ. Since simulated data under the basic coalescent and the two investigated extensions presumed no recombination, IMPUTE2 was run assuming a recombination rate of *ρ* = 0 cM/Mb in simulation experiments. In the baseline simulation scenario, IMPUTE2 was run 10 times using different seeds.

As described above, real data comparisons rested upon a 20 kb chromosomal region with relatively low recombination probability, specifically 0.001% per generation. IMPUTE2 was applied to two different sets of variant sites which represented 10 % of all sites in the investigated region. The first set included only compatible variants. IMPUTE2 was applied to this set assuming no recombination. Results from this set of variants, denominated ‘IMPUTE2 without recombination’ in the following sections, provide the basis for a theoretical comparison with coalescent-based imputation in small chromosomal regions with negligible recombination. The second set included both compatible and incompatible variants. IMPUTE2 was applied to the second variant set incorporating the regional recombination rates provided by the 1000 GP. ‘IMPUTE2 with recombination’ results permit to examine the applicability of the investigated coalescent-based imputation approach to real practice.

The imputation accuracy was primarily quantified by the genotype concordance, defined as the rate of correctly imputed genotypes over all study individuals and all imputed SNPs. The imputation quality score (IQS), which corrects for expected by-chance agreement according to minor allele frequency (MAF), was also calculated [15]. Although the IQS adjusts for genotype agreement by chance, we base our result description on concordance rates, which have a more intuitive interpretation that a Cohen’s kappa coefficient. Means of concordance rate and IQS with corresponding 95% confidence intervals (95% CIs) assuming normality were reported. Results were additionally stratified by MAF to separately examine rare (MAF ≤ 0.01), low-frequency (0.01 < MAF ≤ 0.05) and common (MAF > 0.05) variants. All monomorphic variants were excluded from analysis.

## Results

**Fig. 2:**
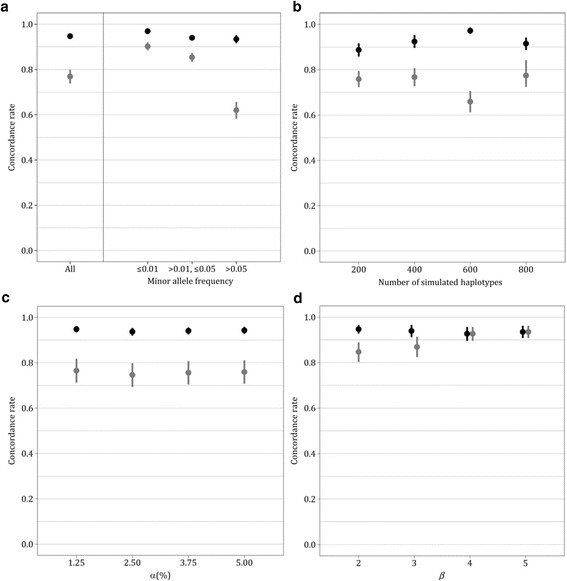
**a-d** Accuracy of imputation relying on the coalescent (black) and with IMPUTE2 (gray) represented by mean concordance rates with the corresponding 95% confidence intervals (shown as error bars). **a** 1000 haplotypes were simulated under the basic coalescent. Genotypes were imputed based on the basic coalescent and with IMPUTE2 without recombination. Results are presented for all variants and stratified by minor allele frequency. **b** Different total numbers of haplotypes were simulated under the basic coalescent. Genotypes were imputed based on the basic coalescent and with IMPUTE2 without recombination. **c** Haplotypes were simulated under the basic coalescent extended to accommodate different exponential growth rates (*α*). Genotypes were imputed based on the coalescent with growth, and with IMPUTE2 without recombination. **d** Haplotypes were simulated under the coalescent extended to consider different numbers of subpopulations (*β*). Genotypes were imputed based on the coalescent with population structure and with IMPUTE2 without recombination

Table 2 shows the number and the MAF distribution of variants simulated under the basic coalescent. In the baseline simulation scenario, the average genotype concordance across all replicates was 0.95 (95%CI: 0.94 to 0.96) for genotype imputation based on the basic coalescent. This method resulted in higher and less variable imputation accuracy than genotype imputation using IMPUTE2, which showed an average genotype concordance of 0.77 (95%CI: 0.74 to 0.81). For illustration, the observed differences in sample means and variances correspond to probability values <0.0001 from both t and F tests. The variability of concordance rates for coalescent-based genotype imputation and IMPUTE2 without recombination was higher among simulation replicates than among iterations with independent selections of measured variant sites (data not shown). For each MAF category, coalescent-based imputation was more accurate than IMPUTE2 (Fig. 2a). The difference in average genotype concordance, and the difference in concordance variability, increased with increasing MAF. Additional file 1: Table S1 shows the corresponding IQS values with analogue findings.

**Table 2:** Distribution of simulated variants according to allele frequency, and dependence of imputation accuracy on the sizes of study population and reference panel. Different total numbers of haplotypes were simulated under the basic coalescent (N_sim_). Genotypes were imputed based on the basic coalescent and with IMPUTE2 without recombination. Mean genotype concordance rates with the corresponding 95% confidence intervals (CIs) are shown for all variants and stratified by minor allele frequency (MAF)

		Variants		Basic coalescent		IMPUTE2	
N_sim_	MAF	N (%)		Mean	(95% CI)	Mean	(95% CI)
*Baseline simulation scenario* ^*a*^							
1000	all	96	(100.0)	0.95	(0.94,0.96)	0.77	(0.74,0.81)
	≤0.01	30	(31.3)	0.97	(0.96,0.98)	0.90	(0.88,0.92)
	>0.01, ≤0.05	24	(25.0)	0.94	(0.93,0.95)	0.85	(0.83,0.87)
	>0.05	42	(43.8)	0.93	(0.92,0.95)	0.62	(0.58,0.66)
*Total number of simulated haplotypes*							
800	all	88	(100.0)	0.91	(0.89,0.94)	0.77	(0.72,0.82)
	≤0.01	27	(30.7)	0.99	(0.98,0.99)	0.99	(0.98,1.00)
	>0.01, ≤0.05	25	(28.4)	0.95	(0.93,0.97)	0.91	(0.89,0.93)
	>0.05	36	(40.9)	0.83	(0.78,0.89)	0.52	(0.47,0.58)
600	all	90	(100.0)	0.97	(0.96,0.99)	0.66	(0.61,0.71)
	≤0.01	15	(16.7)	0.99	(0.98,1.00)	0.98	(0.97,0.99)
	>0.01, ≤0.05	14	(15.6)	0.93	(0.91,0.95)	0.92	(0.89,0.94)
	>0.05	61	(67.8)	0.98	(0.96,1.00)	0.52	(0.49,0.55)
400	all	87	(100.0)	0.92	(0.90,0.95)	0.77	(0.73,0.81)
	≤0.01	19	(21.8)	0.98	(0.97,0.99)	0.97	(0.96,0.98)
	>0.01, ≤0.05	28	(32.2)	0.95	(0.93,0.96)	0.88	(0.83,0.92)
	>0.05	40	(46.0)	0.88	(0.82,0.94)	0.59	(0.56,0.62)
200	all	89	(100.0)	0.89	(0.86,0.92)	0.76	(0.72,0.79)
	≤0.01	10	(11.2)	0.97	(0.96,1.00)	0.97	(0.96,0.99)
	>0.01, ≤0.05	28	(31.5)	0.91	(0.89,0.94)	0.90	(0.88,0.93)
	>0.05	51	(57.3)	0.85	(0.81,0.90)	0.64	(0.61,0.67)

^a^Results averaged over ten simulation replicates and ten iterations with independent selection of measured variant sites

For every simulated population size, coalescent-based genotype imputation achieved higher and less variable concordance rates than IMPUTE2 (Table 2). No monotonic trend of concordance rates was observed with increasing number of simulated haplotypes (Fig. 2b). Most simulated variants had a MAF over 0.05 for any population size. The largest differences between coalescent-based imputation and IMPUTE2 in average genotype concordance, and in concordance variability, were consistently found for this MAF category.

The first investigated generalization of the basic coalescent was population growth (Table 3, Fig. 2c). Including population growth in haplotype simulation resulted in slightly lower concordance rates for coalescent-based genotype imputation and IMPUTE2. No trend in genotype concordance was observed with increasing growth rates. Consideration of population growth in the estimation of gene genealogies by the coalescent-based approach did not improve imputation accuracy. The coalescent-based method, with and without consideration of growth, resulted in higher genotype concordances and higher IQSs than IMPUTE2 in the category of common SNPs (Tables 3 and Additional file 1: Table S2). For rare variants, the advantage or disadvantage of coalescent-based imputation depended on the growth rate, and on the considered accuracy statistic.

**Table 3:** Distribution of simulated variants according to allele frequency, and dependence of imputation accuracy on population growth and structure. 1000 haplotypes were simulated under the coalescent with different exponential growth rates (*α*) and numbers of subpopulations (*β*). Genotypes were imputed based on the basic coalescent, the coalescent incorporating population growth and/or structure and with IMPUTE2 without recombination. Mean genotype concordance rates with the corresponding 95% confidence intervals (CIs) are presented. Results are shown for all variants and stratified by minor allele frequency (MAF)

			Variants		Basic coalescent		Coalescent with growth and/or		IMPUTE2	
*α* (%)	*β*	MAF	N (%)		Mean	(95% CI)	Mean	(95% CI)	Mean	(95% CI)
*Population growth*										
1.25	1	all	87	(100.0)	0.95	(0.94,0.96)	0.95	(0.93,0.96)	0.77	(0.72,0.82)
		≤0.01	30	(34.5)	0.99	(0.98,1.00)	0.99	(0.98,1.00)	0.99	(0.98,1.00)
		>0.01, ≤0.05	20	(23.0)	0.90	(0.88,0.92)	0.90	(0.88,0.92)	0.90	(0.88,0.92)
		>0.05	37	(42.5)	0.94	(0.91,0.97)	0.94	(0.91,0.97)	0.51	(0.46,0.57)
2.50	1	all	86	(100.0)	0.94	(0.92,0.96)	0.94	(0.92,0.95)	0.76	(0.71,0.81)
		≤0.01	25	(29.1)	0.98	(0.97,0.99)	0.98	(0.97,0.99)	0.98	(0.97,0.99)
		>0.01, ≤0.05	23	(26.7)	0.90	(0.88,0.92)	0.90	(0.88,0.92)	0.89	(0.87,0.91)
		>0.05	38	(44.2)	0.93	(0.89,0.96)	0.93	(0.89,0.96)	0.50	(0.45,0.55)
3.75	1	all	88	(100.0)	0.94	(0.93,0.96)	0.94	(0.93,0.96)	0.76	(0.70,0.81)
		≤0.01	27	(31.0)	0.98	(0.97,0.99)	0.98	(0.97,0.99)	0.98	(0.98,0.99)
		>0.01, ≤0.05	24	(27.3)	0.91	(0.89,0.92)	0.91	(0.89,0.92)	0.89	(0.88,0.91)
		>0.05	37	(42.0)	0.93	(0.90,0.96)	0.93	(0.90,0.96)	0.50	(0.45,0.56)
5.00	1	all	88	(100.0)	0.94	(0.93,0.96)	0.94	(0.93,0.96)	0.76	(0.71,0.81)
		≤0.01	27	(30.7)	0.99	(0.98,1.00)	0.99	(0.98,1.00)	0.99	(0.98,1.00)
		>0.01, ≤0.05	24	(27.3)	0.92	(0.90,0.93)	0.92	(0.90,0.93)	0.90	(0.88,0.92)
		>0.05	37	(42.0)	0.93	(0.90,0.96)	0.93	(0.90,0.96)	0.50	(0.45,0.56)
*Population structure*										
0.00	2	all	86	(100.0)	0.95	(0.93,0.97)	0.95	(0.93,0.97)	0.85	(0.81,0.89)
		≤0.01	38	(44.2)	0.96	(0.93,1.00)	0.96	(0.93,1.00)	0.98	(0.97,0.99)
		>0.01, ≤0.05	32	(37.2)	0.94	(0.92,0.95)	0.94	(0.92,0.95)	0.89	(0.88,0.91)
		>0.05	16	(18.6)	0.93	(0.86,0.99)	0.92	(0.86,0.99)	0.44	(0.41,0.48)
0.00	3	all	83	(100.0)	0.94	(0.91,0.97)	0.94	(0.91,0.97)	0.87	(0.82,0.91)
		≤0.01	47	(56.6)	0.97	(0.94,1.00)	0.97	(0.94,1.00)	0.99	(0.98,1.00)
		>0.01, ≤0.05	20	(24.1)	0.92	(0.90,0.94)	0.92	(0.90,0.94)	0.91	(0.89,0.94)
		>0.05	16	(19.3)	0.86	(0.77,0.95)	0.86	(0.77,0.95)	0.47	(0.40,0.53)
0.00	4	all	86	(100.0)	0.93	(0.90,0.96)	0.93	(0.90,0.96)	0.92	(0.88,0.95)
		≤0.01	56	(65.1)	0.97	(0.94,1.00)	0.97	(0.94,1.00)	0.99	(0.98,1.00)
		>0.01, ≤0.05	18	(20.9)	0.95	(0.93,0.97)	0.95	(0.93,0.97)	0.91	(0.89,0.94)
		>0.05	12	(14.0)	0.67	(0.60,0.75)	0.68	(0.60,0.75)	0.59	(0.48,0.70)
0.00	5	all	86	(100.0)	0.94	(0.91,0.96)	0.94	(0.91,0.96)	0.94	(0.92,0.96)
		≤0.01	48	(55.8)	0.97	(0.93,1.00)	0.97	(0.93,1.00)	0.99	(0.98,1.00)
		>0.01, ≤0.05	30	(34.9)	0.93	(0.92,0.94)	0.93	(0.92,0.94)	0.93	(0.91,0.94)
		>0.05	8	(9.3)	0.76	(0.63,0.89)	0.76	(0.63,0.89)	0.67	(0.58,0.76)
*Population growth and structure*										
5.00	5	all	85	(100.0)	0.93	(0.91,0.96)	0.93	(0.90,0.96)	0.94	(0.92,0.96)
		≤0.01	47	(55.3)	0.97	(0.93,1.00)	0.97	(0.93,1.00)	0.99	(0.98,1.00)
		>0.01, ≤0.05	28	(32.9)	0.93	(0.92,0.94)	0.93	(0.92,0.94)	0.93	(0.92,0.94)
		>0.05	10	(11.8)	0.76	(0.67,0.85)	0.76	(0.67,0.85)	0.72	(0.65,0.79)

The second investigated extension of the basic coalescent was population structure (Table 3, Fig. 2d). The largest difference in average genotype concordance between coalescent-based and IMPUTE2 imputation was found for two subpopulations. The number of subpopulations did not practically influence the concordance rates for coalescent-based imputation. By contrast, the average concordance of genotypes imputed by IMPUTE2 increased with increasing number of subpopulations. This result was attributable to the increasing proportion of rare variants with increasing number of subpopulations. Consideration of population structure in the estimation of gene genealogies by the coalescent-based approach did not improve imputation accuracy. As expected due to the small influence of population growth, results for five subpopulations with and without growth were quite similar.

Table 4 presents average accuracies from real data analyses. The MAF distribution of variants imputed based on compatible variants was similar to the distribution of variants imputed based on a set of random measured (compatible and incompatible) variants. In the hypothetical European (CEU) and African (AFR) studies, most variants showed a low-frequency (0.01 < MAF ≤ 0.05). In the hypothetical study in Latin America (AMR), rare SNPs (MAF ≤ 0.01) built the largest variant category.

**Table 4:** Distribution of simulated variants according to allele frequency, and accuracy of variants imputed based on the basic coalescent, with IMPUTE2 without recombination, and with IMPUTE2 with recombination. Real genotypes were retrieved from the 1000 Genomes Project (1000 GP) assuming that the CEU, AFR and AMR subpopulations constituted hypothetical study populations. Remaining 1000 GP individuals built the external reference panel for genotype imputation. Mean concordance rates with the corresponding 95% confidence intervals (CIs) are represented. Results are shown for all variants and also stratified by minor allele frequency (MAF)

		Variants		Basic coalescent		IMPUTE2 without recombination		Variants		IMPUTE2 with recombination	
Study	MAF	N (%)		Mean	(95% CI)	Mean	(95% CI)	N (%)		Mean	(95% CI)
CEU	all	41	(100.0)	0.92	(0.89,0.95)	0.90	(0.86,0.93)	42	(100.0)	0.93	(0.89,0.96)
	≤0.01	11	(26.8)	0.98	(0.97,0.99)	0.98	(0.97,0.99)	12	(28.6)	0.98	(0.97,0.99)
	>0.01, ≤0.05	21	(51.2)	0.92	(0.91,0.93)	0.91	(0.91,0.92)	19	(45.2)	0.99	(0.97,1.00)
	>0.05	9	(22.0)	0.84	(0.70,0.99)	0.75	(0.62,0.88)	11	(26.2)	0.76	(0.68,0.85)
AFR	all	123	(100.0)	0.93	(0.92,0.95)	0.93	(0.91,0.95)	121	(100.0)	0.96	(0.94,0.97)
	≤0.01	35	(28.5)	0.99	(0.98,1.00)	0.99	(0.98,1.00)	37	(30.6)	0.99	(0.98,1.00)
	>0.01, ≤0.05	58	(47.2)	0.97	(0.96,0.98)	0.97	(0.96,0.97)	56	(46.3)	0.96	(0.95,0.97)
	>0.05	30	(24.4)	0.81	(0.76,0.85)	0.79	(0.74,0.83)	28	(23.1)	0.90	(0.85,0.95)
AMR	all	66	(100.0)	0.92	(0.90,0.95)	0.92	(0.89,0.94)	67	(100.0)	0.96	(0.94,0.98)
	≤0.01	36	(54.5)	0.98	(0.97,0.99)	0.98	(0.97,0.99)	36	(53.7)	0.99	(0.98,1.00)
	>0.01, ≤0.05	5	(7.6)	0.91	(0.88,0.94)	0.90	(0.86,0.95)	7	(10.4)	0.93	(0.90,0.96)
	>0.05	25	(37.9)	0.84	(0.79,0.89)	0.82	(0.76,0.87)	24	(35.8)	0.93	(0.87,0.98)

CEU…Utah residents with Northern and Western European ancestry; AFR…African populations including African Ancestry in Southwest US (ASW), Luhya in Webuye, Kenya (LWK) and Yoruba in Ibadan, Nigeria (YRI); AMR…American populations including Colombian in Medellin, Colombia (CLM), Mexican Ancestry in Los Angeles, California (MXL) and Puerto Rican in Puerto Rico (PUR)

In the hypothetical European study, imputation based on the coalescent, imputation with IMPUTE2 without recombination, and imputation with IMPUTE2 incorporating 1000 GP recombination rates achieved similar average concordance rates. In the hypothetical studies in Africa (AFR) and Latin America (AMR), IMPUTE2 with recombination slightly outperformed coalescent-based imputation. Similar conclusions can be drawn based on IQS values (Additional file 1: Table S3).

## Discussion

This article describes the development of a novel method for imputation of genotypes relying on the basic coalescent, which was extended to consider population growth and structure. Standard methods for genotype imputation take advantage of present LD patterns. By contrast, coalescent-based imputation capitalizes on the process which originated current haplotype structure. Our simulations revealed that, in LD-blocks, the concordance of genotypes imputed under the basic coalescent was higher and less variable than for IMPUTE2 independently of the size of the study and reference populations. Explicit consideration of population growth did not practically improve imputation accuracy, even if growth was present. Nordborg suggests that although population growth has clearly taken place during the last 200,000 years, it may be reasonable to ignore it when modelling human evolution [16]. Genealogies coalesce earlier in history for growing than for constant-size populations, but this is hardly relevant in an imputation context where templates are identified based on the ranking rather than actual coalescence times.

Our simulations also suggested that the advantage of coalescent-based over standard imputation increased with the allele frequency and decreased with population stratification. The increasing proportion of rare variants with an increasing number of subpopulations probably explains the similar performance of genotype imputation based on the coalescent and with IMPUTE2 in the simulated scenario with five subpopulations. To date, coalescent-based imputation is computationally much more demanding than IMPUTE2. For example, one imputation run based on the coalescent required about 6.5 h for simulated haplotypes in a UNIX based high performance computing cluster. The same run on the sample cluster took 30 min for IMPUTE2, with and without recombination.

The basic coalescent was initially developed in the 1980s [17]. One decade later, it was generalized in order to accommodate populations with a demographic history of growth and subdivision [6, 7, 18, 19]. Kimmel et al. proposed in the late 2000s a genealogy-based algorithm for fine-mapping, which permitted to evaluate the significance and location of putative causal variants [20]. Paşaniuc et al. applied the coalescent to select personalized reference panels for genotype imputation [21]. They proposed to select reference individuals based on weights inversely proportional to the coalescence time between study and reference haplotypes and genotype imputation was actually carried out with standard methods, concretely with IMPUTE2. The methodology introduced in the present article is substantially different from the techniques proposed by Paşaniuc et al. regarding both the use of coalescence times to identify imputation templates, and the actual imputation of missing allele states in study individuals. In spite of these differences and in line with our real data results, Paşaniuc et al. also found that coalescent-based techniques may offer some advantage for genotype imputation, in particular for genetic regions of low LD. Jewett et al. relied on a two-population model to investigate the dependence of imputation accuracy on the size of the reference panel and on the coalescence time of study and reference haplotypes [22]. The same two-population model was assumed by Huang et al., who derived approximate expressions for the imputation accuracy as a function of population-genetic factors including the mutation rate [23]. The focus of this article is markedly different. Here we developed and tested a novel procedure to impute genotypes based on the coalescent, and applied the new method to simulated and real datasets. Jewett et al. found that a small reference panel from the same population as study individuals yields higher imputation accuracy than a large reference panel from a markedly different population. Our results complement this finding and indicate that the relationship between the size of the study and reference populations and imputation accuracy is complex.

The effective population size, mutation rate and sequence length that were assumed to simulate haplotypes under the coalescent are comparable to the parameter setting in other simulation experiments. Fu et al. investigated effective population sizes up to 8000, a mutation rate of 1.5 × 10^−8^ per site per generation, sequence lengths of up to 50 kb and growth rates of up to 2.5% per generation [24]. Kang and Marjoram simulated 1000 haplotypes over a 500 kb region assuming a scaled mutation rate of 100 [25]. Zhang et al. imputed 100 kb in 2000 simulated haplotypes of 1 Mb total length and used an effective population size of 10,000 and a mutation rate of 10^−8^ per site per generation as simulation parameters [26]. The time points for population growth and subdivision selected in present simulation experiments are also conform to established models for human demography, e.g. the Out-of-Africa model [27]. Therein, the Eurasian population, which developed through a bottle-neck from the African population, split into the Asian, American and European subpopulations approximately 50,000 years ago. The starting point of exponential population growth was set to a later time point. Here, 5000 years seemed reasonable, because intense agriculture involving various cultivated crops and the domestication of farm animals had developed throughout Asia, Europe and America.

We constrained our investigations to LD-blocks. Even if genotypes are imputed in practice over the whole genome, recombination breaks allelic association and imputed genotypes essentially depend on measured genotypes in the same LD-block. LD-block confinement permitted to estimate gene genealogies under the assumption that genetic variability was completely due to mutation without recombination. This assumption is essential for estimating perfect genealogical trees. Analyses of real European data showed similar accuracies for coalescent-based and standard imputation. Only compatible sites according to pairwise four-gamete tests were selected as directly measured in order to be able to represent real data, which naturally comprises variants originated from recombination, by single trees. This was accompanied by some computational complexity and selected variants unlikely represented the complete genetic variability, which could be one reason of the relatively weak performance of coalescent-based and IMPUTE2 without recombination imputation methods for real African and Latin American data. For these two scenarios which represent hypothetical studies with genetically diverse and admixed individuals, respectively, imputation accuracy improved after incorporating estimates of recombination based on a set of randomly selected measured SNP sites. When IMPUTE2 was run incorporating estimates of recombination based on a set of randomly selected measured SNP sites, the imputation accuracy was clearly superior to coalescent-based imputation. Hence, the difference in imputation accuracy between coalescent-based and standard imputation for real data might be due to the inclusion of variants created through recombination. A lot of effort has been made to incorporate recombination in coalescent-based methods [11]. In theory, ancestral recombination graphs (ARGs) can be used to model the ancestral relationship between haplotypes, which include nodes for recombination in addition to coalescence nodes. Due to the very complex nature of this problem, the construction of ARGs is extremely difficult in practice. Often the search for possible ARGs is computationally intractable and there is not enough information in the data to reconstruct an ARG with adequate confidence [28]. Present findings clearly illustrate the potential of coalescent-based genotype imputation and emphasize the urgent need for feasible solutions to incorporate recombination in coalescence inference.

## Conclusions

Coalescent-based genotype imputation within LD-blocks showed a better accuracy than standard imputation in simulations with variable population sizes, with and without population growth and with few subpopulations. However, standard imputation is computationally much less demanding. To exploit the full potential of coalescent-based methods for the imputation of missing genotypes in genetic studies, further methodological research is needed to reduce computer time, to take into account recombination, and to implement these methods in user-friendly computer programs. The contribution of the present investigation in this direction is twofold. First, we provide computer code which takes advantage of publicly available software in order to impute genotypes relying on the basic coalescent and two extensions thereof. Second, we use simulations and real 1000 GP data to explore the potential of this alternative approach with a focus on the accuracy of imputed genotypes. Both the provided material and reported findings shall boost methodological research and the use of coalescent methods in general, and of coalescent-based genotype imputation in particular.

## Additional files

Additional file 1: Table S1. Dependence of imputation accuracy (mean imputation quality scores (IQS) with corresponding 95% confidence intervals) on the sizes of study population and reference panel. An IQS equal to 1 can be interpreted as 100% imputation accuracy, an IQS of 0 corresponds to random genotype assignment, and negative IQS points to a genotype assignment worse than by chance. **Table S2.** Dependence of imputation accuracy on population growth and structure (mean imputation quality scores (IQS) with corresponding 95% confidence intervals). **Table S3.** Accuracy of variants imputed based on the basic coalescent, with IMPUTE2 without recombination, and with IMPUTE2 with recombination (mean imputation quality scores (IQS) with corresponding 95% confidence intervals). (DOCX 39 kb)

## Abbreviations

1000 GP: 1000 Genomes Project
AFR: African populations
AMR: American populations
ARG: Ancestral recombination graph
ASW: African Ancestry in Southwest US
CEU: Utah residents with Northern and Western European ancestry
CLM: Colombian in Medellin, Colombia
cm/kb: centimorgan per megabase
DNA: deoxyribonucleic acid
IQS: Imputation quality score
LD: Linkage disequilibrium
LWK: Luhya in Webuye, Kenya
MAF: Minor allele frequency
MXL: Mexican Ancestry in Los Angeles, California
PUR: Puerto Rican in Puerto Rico
SNP: Single nucleotide polymorphism
YRI: Yoruba in Ibadan, Nigeria
