# Supplementary material for: Imputation of missing genotypes within LD-blocks relying on the basic coalescent and beyond: consideration of population growth and structure
Source: BMC Genomics. 2017 Oct 17;18:798. doi: 10.1186/s12864-017-4208-2 (PMC5646149; doi:10.1186/s12864-017-4208-2)
Supplement: Supplementary file 1 — Dependence of imputation accuracy (mean imputation quality scores (IQS) with corresponding 95% confidence intervals) on the sizes of study population and reference panel. An IQS equal to 1 can be interpreted as 100% imputation accuracy, an IQS of 0 corresponds to random genotype assignment, and negative IQS points to a genotype assignment worse than by chance. Table S2. Dependence of imputation accuracy on population growth and structure (mean imputation quality scores (IQS) with corresponding 95% confidence intervals). Table S3. Accuracy of variants imputed based on the basic coalescent, with IMPUTE2 without recombination, and with IMPUTE2 with recombination (mean imputation quality scores (IQS) with corresponding 95% confidence intervals). (DOCX 39 kb) [file 12864_2017_4208_MOESM1_ESM.docx]

Imputation of Missing Genotypes in LD-Blocks Relying on the Basic Coalescent and Beyond: Consideration of Population Growth and Structure

Maria Kabisch^1,2^, Ute Hamann^1^, Justo Lorenzo Bermejo^2^*

^1^ Molecular Genetics of Breast Cancer, German Cancer Research Center (DKFZ), Heidelberg, 69120, Germany

^2^ Institute of Medical Biometry and Informatics, University Hospital Heidelberg, Heidelberg, 69120, Germany

* To whom correspondence should be addressed:

E-mail: lorenzo@imbi.uni-heidelberg.de

Telephone: +49-(0)6221 56 4180

Telefax: +49-(0)6221 56 4195

**Table S1** Dependence of imputation accuracy (mean imputation quality scores (IQS) with corresponding 95% confidence intervals) on the sizes of study population and reference panel. An IQS equal to 1 can be interpreted as 100% imputation accuracy, an IQS of 0 corresponds to random genotype assignment, and negative IQS points to a genotype assignment worse than by chance.

|  |  | Basic coalescent | | | IMPUTE2 without recombination | | | |
| --- | --- | --- | --- | --- | --- | --- | --- | --- |
| N_sim_ | MAF | Mean | | (95% CI) | Mean | | (95% CI) | |
| *Baseline simulation scenario^a^* | | | | | | | | |
| 1,000 | all | 0.40 | (0.35,0.46) | | -0.01 | (-0.05,0.04) | |  |
|  | ≤0.01 | 0.26 | (0.16,0.36) | | 0.04 | (-0.09,0.16) | |  |
|  | >0.01, ≤0.05 | 0.28 | (0.18,0.38) | | -0.04 | (-0.13,0.05) | |  |
|  | >0.05 | 0.59 | (0.53,0.66) | | -0.02 | (-0.06,0.02) | |  |
| *Total number of simulated haplotypes* | | | | | | | | |
| 800 | all | 0.39 | (0.27,0.51) | | 0.00 | (-0.08,0.09) | |  |
|  | ≤0.01 | 0.12 | (-0.13,0.38) | | -0.03 | (-0.29,0.23) | |  |
|  | >0.01, ≤0.05 | 0.44 | (0.24,0.65) | | 0.05 | (-0.07,0.18) | |  |
|  | >0.05 | 0.56 | (0.41,0.70) | | -0.01 | (-0.05,0.04) | |  |
| 600 | all | 0.74 | (0.66,0.82) | | 0.06 | (0.03,0.10) | |  |
|  | ≤0.01 | 0.32 | (0.12,0.52) | | 0.13 | (-0.07,0.32) | |  |
|  | >0.01, ≤0.05 | 0.30 | (0.15,0.45) | | 0.18 | (0.08,0.28) | |  |
|  | >0.05 | 0.94 | (0.89,0.99) | | 0.02 | (0.01,0.03) | |  |
| 400 | all | 0.44 | (0.30,0.57) | | -0.02 | (-0.11,0.08) | |  |
|  | ≤0.01 | 0.08 | (-0.33,0.49) | | -0.01 | (-0.35,0.33) | |  |
|  | >0.01, ≤0.05 | 0.25 | (0.04,0.47) | | -0.16 | (-0.35,0.03) | |  |
|  | >0.05 | 0.73 | (0.60,0.87) | | 0.08 | (0.06,0.11) | |  |
| 200 | all | 0.42 | (0.29,0.54) | | -0.02 | (-0.10,0.07) | |  |
|  | ≤0.01 | -0.02 | (-0.82,0.79) | | -0.13 | (-0.71,0.45) | |  |
|  | >0.01, ≤0.05 | 0.73 | (0.04,0.36) | | 0.06 | (-0.13,0.25) | |  |
|  | >0.05 | 0.62 | (0.51,0.73) | | -0.04 | (-0.07,0.00) | |  |

^a^ Results averaged over ten simulation replicates and ten iterations with independent selection of measured variant sites

**Table S2** Dependence of imputation accuracy on population growth and structure (mean imputation quality scores (IQS) with corresponding 95% confidence intervals).

|  |  | |  | Basic coalescent | | | Coalescent with  growth and/or  structure | | | | IMPUTE2 without recombination | | |  |  |
| --- | --- | --- | --- | --- | --- | --- | --- | --- | --- | --- | --- | --- | --- | --- | --- |
| *α* (%) | *β* | | MAF | Mean | | (95% CI) | Mean | | (95% CI) | | Mean | | (95% CI) |  |  |
| *Population growth* | | | | | | | | | | | | | |  |  |
| 1.25 | 1 | all | | 0.29 | (0.11-0.47) | | 0.29 | (0.11-0.47) | | -0.09 | | (-0.25-0.07) | |  |  |
|  |  | ≤0.01 | | -0.18 | (-0.59-0.24) | | -0.18 | (-0.59-0.24) | | -0.29 | | (-0.29-0.16) | |  |  |
|  |  | >0.01, ≤0.05 | | 0.08 | (-0.08-0.23) | | 0.08 | (-0.08-0.23) | | 0.01 | | (-0.14-0.17) | |  |  |
|  |  | >0.05 | | 0.78 | (0.67-0.89) | | 0.78 | (0.67-0.89) | | 0.01 | | (-0.01-0.03) | |  |  |
| 2.50 | 1 | all | | 0.32 | (0.16-0.47) | | 0.32 | (0.16-0.47) | | -0.08 | | (-0.20-0.05) | |  |  |
|  |  | ≤0.01 | | -0.20 | (-0.57-0.17) | | -0.20 | (-0.57-0.17) | | -0.27 | | (-0.70-0.15) | |  |  |
|  |  | >0.01, ≤0.05 | | 0.09 | (-0.06-0.25) | | 0.09 | (-0.06-0.25) | | -0.01 | | (-0.14-0.13) | |  |  |
|  |  | >0.05 | | 0.79 | (0.68-0.90) | | 0.79 | (0.68-0.90) | | 0.01 | | (-0.01-0.03) | |  |  |
| 3.75 | 1 | all | | 0.34 | (0.19-0.49) | | 0.34 | (0.19-0.49) | | -0.02 | | (-0.14-0.09) | |  |  |
|  |  | ≤0.01 | | -0.09 | (-0.49-0.31) | | -0.09 | (-0.49-0.31) | | -0.12 | | (-0.51-0.27) | |  |  |
|  |  | >0.01, ≤0.05 | | 0.16 | (0.10-0.21) | | 0.16 | (0.10-0.21) | | 0.04 | | (-0.03-0.12) | |  |  |
|  |  | >0.05 | | 0.77 | (0.65-0.89) | | 0.77 | (0.66-0.89) | | 0.01 | | (-0.01-0.03) | |  |  |
| 5.00 | 1 | all | | 0.31 | (0.15-0.47) | | 0.31 | (0.15-0.47) | | -0.06 | | (-0.19-0.07) | |  |  |
|  |  | ≤0.01 | | -0.17 | (-0.59-0.25) | | -0.17 | (-0.59-0.25) | | 0.13 | | (-0.65-0.19) | |  |  |
|  |  | >0.01, ≤0.05 | | 0.14 | (0.04-0.23) | | 0.14 | (0.04-0.23) | | 0.06 | | (-0.09-0.13) | |  |  |
|  |  | >0.05 | | 0.77 | (0.65-0.89) | | 0.77 | (0.65-0.89) | | 0.01 | | (-0.01-0.03) | |  |  |
| *Population structure* | | | | | | | | | | | | | | |  |
| 0.00 | 2 | | all | 0.29 | (0.11,0.46) | | 0.28 | (0.11,0.46) | | -0.09 | | (-0.25,0.07) | |  |  |
|  |  | | ≤0.01 | 0.05 | (-0.29,0.39) | | 0.04 | (-0.30,0.39) | | -0.15 | | (-0.50,0.21) | |  |  |
|  |  | | >0.01, ≤0.05 | 0.29 | (0.12,0.45) | | 0.29 | (0.12,0.45) | | -0.06 | | (-0.16,0.05) | |  |  |
|  |  | | >0.05 | 0.84 | (0.70,0.99) | | 0.85 | (0.70,0.99) | | -0.02 | | (-0.02,0.01) | |  |  |
| 0.00 | 3 | | all | 0.21 | (0.05,0.38) | | 0.21 | (0.05,0.38) | | -0.07 | | (-0.23,0.08) | |  |  |
|  |  | | ≤0.01 | 0.12 | (-0.15,0.39) | | 0.12 | (-0.15,0.39) | | -0.11 | | (-0.38,0.17) | |  |  |
|  |  | | >0.01, ≤0.05 | 0.07 | (-0.09,0.23) | | 0.07 | (-0.09,0.23) | | -0.05 | | (-0.24,0.14) | |  |  |
|  |  | | >0.05 | 0.68 | (0.46,0.89) | | 0.68 | (0.46,0.89) | | -0.01 | | (-0.05,0.05) | |  |  |
| 0.00 | 4 | | all | 0.17 | (0.01,0.32) | | 0.16 | (0.00,0.32) | | -0.03 | | (-0.19,0.13) | |  |  |
|  |  | | ≤0.01 | 0.07 | (-0.16,0.30) | | 0.06 | (-0.17,0.30) | | -0.06 | | (-0.31,0.19) | |  |  |
|  |  | | >0.01, ≤0.05 | 0.42 | (0.21,0.62) | | 0.44 | (0.23,0.64) | | 0.02 | | (-0.12,0.15) | |  |  |
|  |  | | >0.05 | 0.22 | (0.13,0.32) | | 0.22 | (0.12,0.32) | | 0.04 | | (-0.03,0.12) | |  |  |
| 0.00 | 5 | | all | -0.02 | (-0.27,0.24) | | -0.02 | (-0.28,0.24) | | 0.01 | | (-0.20,0.22) | |  |  |
|  |  | | ≤0.01 | -0.09 | (-0.54,0.36) | | -0.10 | (-0.56,0.36) | | 0.03 | | (-0.35,0.40) | |  |  |
|  |  | | >0.01, ≤0.05 | 0.03 | (-0.07,0.13) | | 0.04 | (-0.07,0.14) | | -0.01 | | (-0.07,0.06) | |  |  |
|  |  | | >0.05 | 0.27 | (-0.03,0.56) | | 0.26 | (-0.03,0.56) | | -0.01 | | (0.07,0.05) | |  |  |
| *Population growth and structure* | | | | | | | | | | | | | | | |
| 5.00 | 5 | | all | -0.04 | (-0.23,0.14) | | -0.05 | (-0.23,0.13) | | -0.03 | | (-0.20,0.13) | |  |  |
|  |  | | ≤0.01 | -0.12 | (-0.45,0.21) | | -0.13 | (-0.46,0.20) | | -0.06 | | (-0.36,0.24) | |  |  |
|  |  | | >0.01, ≤0.05 | 0.01 | (-0.09,0.10) | | 0.01 | (-0.09,0.10) | | -0.01 | | (-0.10,0.09) | |  |  |
|  |  | | >0.05 | 0.18 | (0.02,0.34) | | 0.19 | (0.02,0.35) | | 0.02 | | (-0.04,0.07) | |  |  |

**Table S3** Accuracy of variants imputed based on the basic coalescent, with IMPUTE2 without recombination, and with IMPUTE2 with recombination (mean imputation quality scores (IQS) with corresponding 95% confidence intervals).

|  |  | Basic coalescent | | IMPUTE2 without  recombination | | IMPUTE2 with  recombination | |
| --- | --- | --- | --- | --- | --- | --- | --- |
| Study | MAF | Mean | (95% CI) | Mean | (95% CI) | Mean | (95% CI) |
| CEU | all | 0.13 | (-0.01,0.27) | 0.00 | (-0.13,0.13) | 0.52 | (0.38,0.66) |
|  | ≤0.01 | 0.22 | (-0.11,0.55) | 0.11 | (-0.23,0.44) | 0.14 | (-0.12,0.41) |
|  | >0.01, ≤0.05 | -0.13 | (-0.22,-0.05) | -0.22 | (-0.31,-0.12) | 0.87 | (0.75,1.00) |
|  | >0.05 | 0.62 | (0.34,0.91) | 0.38 | (0.17,0.59) | 0.32 | (0.22,0.42) |
| AFR | all | 0.28 | (0.12,0.43) | 0.30 | (0.17,0.42) | 0.35 | (0.20,0.49) |
|  | ≤0.01 | -0.10 | (-0.64,0.44) | 0.05 | (-0.38,0.48) | -0.03 | (-0.47,0.40) |
|  | >0.01, ≤0.05 | 0.46 | (0.43,0.50) | 0.45 | (0.41,0.49) | 0.43 | (0.38,0.48) |
|  | >0.05 | 0.36 | (0.26,0.45) | 0.29 | (0.22,0.36) | 0.68 | (0.56,0.80) |
| AMR | all | 0.22 | (0.08,0.36) | 0.11 | (-0.01,0.24) | 0.37 | (0.23,0.51) |
|  | ≤0.01 | 0.14 | (-0.10,0.37) | 0.00 | (-0.22,0.21) | 0.14 | (-0.07,0.34) |
|  | >0.01, ≤0.05 | 0.05 | (-0.55,0.64) | -0.04 | (-0.68,0.60) | 0.29 | (0.06,0.51) |
|  | >0.05 | 0.37 | (0.27,0.47) | 0.31 | (0.24,0.38) | 0.75 | (0.62,0.88) |

CEU…Utah residents with Northern and Western European ancestry; AFR…African populations including African Ancestry in Southwest US (ASW), Luhya in Webuye, Kenya (LWK) and Yoruba in Ibadan, Nigeria (YRI); AMR…American populations including Colombian in Medellin, Colombia (CLM), Mexican Ancestry in Los Angeles, California (MXL) and Puerto Rican in Puerto Rico (PUR).
